# Authors' reply

**DOI:** 10.1192/bjb.2021.79

**Published:** 2021-10

**Authors:** Lucy Griffin, Katie Clyde, Richard Byng, Susan Bewley

**Affiliations:** Psychiatrist, Royal College of Psychiatrists, UK; Email: drlucygriffin@outlook.com; Psychiatrist, Royal College of Psychiatrists, UK; Psychiatrist, Royal College of Psychiatrists, UK; Psychiatrist, Royal College of Psychiatrists, UK

Thank you for publishing our article^1^ and facilitating academic debate in this rapidly evolving area of healthcare. It is unsurprising to find disagreement over the interpretation of existing research, and we welcome this opportunity to respond to Ashley and review the many references supplied, including data published after our article was accepted. We happily address Ashley's points and respond to all individual references in Table 1 https://doi.org/10.6084/m9.figshare.13626380.v3.

We stated that the College sees the placement of barriers to seeking transition as a form of conversion therapy, and we shortened – but did not substantively change – the position statement quote.^2^ We believe it accurately represents the meaning, and indeed the same abbreviation was used within the body of the position statement itself. If we are wrong about the College's stance, and we agree there is a lack of specificity about barriers, others too may be confused. Given moves to criminalise conversion therapies,^3^ the College should clarify what constitutes unacceptable practice^4^ and be clear that provision of psychological interventions to address existing mental health needs before referral for cross-sex hormones, or surgery, would not constitute a barrier or conversion therapy. This is of particular importance since the recent UK judicial review, which found that young people are unlikely to be able to provide informed consent for early medical intervention.

We agree we used some non-peer-reviewed literature. It is well established that dissenting voices can go unheard by invested clinicians and reports of harm take much longer to recognise.^5^ In a word-constrained, broad-based discussion, we could not analyse all the existing literature about natural history, persistence of gender incongruence in youth, rising referrals, or the age and sex switches, though these have been covered elsewhere.^6^ Similarly, other authors have noted that gender dysphoria is a common step in the developmental pathway of same-sex attraction.^7^

Proponents of affirmative care often claim that medical transition is well studied, with academic consensus on effectiveness. In reality, the literature is fraught with study design problems, including convenience sampling, lack of controls, small sample sizes, short study lengths and high drop-out rates among participants. Most of the studies cited in Ashley's letter were of cross-sectional observational design (Table 1) https://doi.org/10.6084/m9.figshare.13626380.v3. These low quality studies are unable to demonstrate causality and are susceptible to confounders. An important example of the shortcomings of such convenience sampling is the 2020 paper by Turban et al, which claims to demonstrate lower suicidal ideation in adults who had been prescribed GnRHa in adolescence.^8^ There are a number of methodological shortcomings associated with this biased sampling,^9^ the most worrying being the authors’ failure to recognise that this single positive finding is inevitable as prescribers would only have offered puberty blockers to adolescents with stable mental health. Those adolescents with severe psychological problems would not have been eligible. Suicidal ideation is almost certainly related to poor mental health (both past and present) in this group rather than any lack of puberty blockade. This same fact also renders any retrospective desire for treatment invalid. The authors’ recommendation that ‘this treatment [should] be made available to transgender adolescents who want it’ is unsupportable.

Before-and-after studies and case-note reviews are similarly unreliable. Like first-hand accounts, they are mainly useful for raising, not answering, quantitative questions. Benefits will be bolstered by mutual belief systems, clinician charisma, powerful mood-altering drugs and body modifications, as well as the placebo effect. By their nature, these kinds of evidence are unconvincing to an ethical medical profession with a long history of causing harm.^5^

The problem of missing data distorts routinely quoted high levels of satisfaction and low regret rates in transgender healthcare. It is unknown whether large losses to follow-up in gender dysphoria research, often over 30%,^10^ mask adverse effects, including death by suicide, cardiovascular disease or general morbidity associated with deteriorating mental and physical health. Thus, long-term cohort data, as well as appropriate randomised trials, are essential.^11^

Publication bias in this area can be demonstrated by a key ‘positive’ published study^12^ which was widely reported by media outlets. This population-based study initially reported a ‘longitudinal association between gender-affirming surgery and reduced likelihood of mental health treatment’ and declared that these findings ‘lend support to the decision to provide gender-affirming surgeries to transgender individuals who seek them’. However, when the authors were asked to address significant methodological limitations and reanalyse including a comparator group, any purported benefit disappeared.^13^ The journal editor stated that the original conclusion of the benefits of surgeries ‘was too strong’, and that the data ‘demonstrated no advantage of surgery in relation to subsequent mood or anxiety disorder-related health care visits or prescriptions or hospitalizations following suicide attempts’.^14^ The published correction garnered much less media interest than the initial flawed research.

We agree with Ashley that scientific literature must meet the highest standard for publication and that competent care depends on the integrity of the scientific process. We would add that gender healthcare deserves the same rigorous scientific underpinning as all other areas of medicine. However, in the absence of double-blind randomised controlled trials, there can be no analysis of metadata. The often cited ‘What we Know’ project does not meet even the minimum standard of a systematic review.^15^ Poor-quality publications are then recycled as ‘evidence’ and can form the basis of poor-quality guidelines,^16^ which in turn are cited as further evidence that this ‘treatment’ works.

Ashley calls for love, but this quality resides outside the consultation room and is not a medical intervention. We support the highest-quality compassionate and evidence-based care for all individuals, based on their own values and circumstances, and are only opposed to bad science^17,18^ which supports, promotes or sells medical interventions without reliably quantifying the outcomes. Present and future patients deserve better, unbiased, sound evidence. Higher-quality collaborative research and independent adjudication of the evidence^19^ are required to find out exactly what works, for whom, when and for how long.
